# 896. Systemwide Burn Care Integration: Advancing Outreach and Education

**DOI:** 10.1093/jbcr/irag033.392

**Published:** 2026-04-06

**Authors:** Haily Smith, Tracie Burchett

**Affiliations:** University of Louisville Health - University of Louisville Hospital, Shelbyville, KY; University of Louisville Burn Center, Louisville, KY

## Abstract

**Introduction:**

Emergency departments in a healthcare system associated with the verified burn center expressed a deficit in burn care and need for increased competency in stabilizing the burn patient. The ED's identified an increasing number of burn-related injuries and patients requiring transfer. The verified burn center additionally desired an increased relationship with the ED's within the system, with a desire to improve burn care. An educational program was developed, with emphasis on increasing the competency and confidence of nursing staff. The burn center worked to provide monthly updates on transfer patients, to continue providing resources and high-quality feedback.

**Methods:**

When registering for the in-service, nursing staff were asked to complete a Likert scale survey measuring their confidence and comfort in caring for the burn patient. Questions asked included general measurements of confidence and specific topics such as estimating burn depth and identifying transfer criteria. Immediately after the education, nursing staff were asked the same questions to gauge effectiveness and understanding. The burn educator discussed several transfer cases with the group and provided follow-up on patients transferred from that facility. Going forward, follow up information and outcomes are sent to the facility on all burn patient transfers, to allow open communication.

**Results:**

Of all five questions asked before and after the in service, respondents reported improvement in confidence when caring for burn patients. Respondents especially showed improvement in comfort estimating burn surface area, burn depth and in caring for the burn injured patient. Follow up information regarding patients transferred from the healthcare system is sent monthly to the department manager, to be shared with staff involved in care. Staff at the outlying facility and the burn educator antidotally report a more open relationship and ability to ask care questions when a patient is transferred.

**Conclusions:**

Improving care and incorporating the ED's in the healthcare system with a verified burn center is a desire by both the burn center and the transferring facilities. As a facility associated with a burn center, the burn educator noted an opportunity to ensure nursing staff were confident and comfortable in all aspects of initial burn care.

**Applicability of Research to Practice:**

This study notes the successful implementation of education to the emergency departments associated with a burn center and one potential way of integrating a healthcare system with the burn center’s approach. Education and outreach to the staff in the associated emergency departments help to bridge educational and communication gaps, improving burn care and the confidence of nurses caring for the patient. The burn center aims to impact burn care throughout its healthcare system, so that any patient seeking care in a facility associated with the burn center can be confident the nursing staff is prepared.

**Funding for the study:**

N/A.

